# Anisotropy Influences on the Drug Delivery Mechanisms by Means of Joint Invariant Functions

**DOI:** 10.1155/2017/5748273

**Published:** 2017-09-10

**Authors:** G. Cioca, E. S. Bacaita, M. Agop, C. Lupascu Ursulescu

**Affiliations:** ^1^Faculty of Medicine, Lucian Blaga University of Sibiu, Victoriei Bld. No. 10, 550024 Sibiu, Romania; ^2^Department of Physics, “Gheorghe Asachi” Technical University of Iasi, Iasi, Romania; ^3^Academy of Romanian Scientists, Bucharest, Romania; ^4^Department of Radiology, “Grigore T. Popa” University of Medicine and Pharmacy, Iasi, Romania

## Abstract

In the frame of Higuchi's type functionality, this paper presents the anisotropy influences on the drug delivery mechanisms through the joint invariant functions to the simultaneous actions of the two SL(2R) isomorphic groups. Then, a new equation for drug delivery mechanism, independent of the type of polymer matrix and/or drug, is proposed.

## 1. Introduction

After administration and distribution, a drug has therapeutic effect if the molecules have affinity and selectivity for its pharmacological target. But in the same time it is very important to realise an optimal concentration interval because concentrations above or below this interval can produce toxic manifestations or no therapeutic effects. The way by which a drug is released from a particular formulation can have a remarkable effect on its efficacy or toxic effects. In this context, the aim is to reduce to minimum the risks of administration by maintaining drug level within a desired range. Following conventional drug administration is possible to avoid high variation of plasma concentration if it used multidose therapy. The interval between doses is calculated using a pharmacokinetic parameter named half-life time of the drug. In the last years, the interdisciplinary research that combines chemistry, pharmacology, and molecular biology released new pharmaceuticals forms which provide a specific quantity of a therapeutic substance for a prolonged period of time to a target area within the body.

One of these forms are the drug delivery systems (DDS), based on biocompatible polymers. Depending on DDS and application type, studies reveled that several phenomena occur simultaneously or concurrently. These phenomena, mentioned in the order of their appearance, are as follows:

- *water diffusion inside DDS* due to water concentration gradient between release environment and DDS;
- *swelling of polymeric matrix* due to the penetration of water, determining an increase of system size, and, as a consequence, also variations in drug concentration inside DDS;
- *drug diffusion out of DDS* due to drug concentration gradient between release environment and DDS; in time, the polymer matrix swelling will determine a more relaxed polymer network for which the mean free path and implicitly the diffusion coefficient of drug particles are higher;
- depending on polymer network density length, at a certain moment, the* polymer matrix itself dissolves* more or less rapidly [1].

In most of the theories developed so far, diffusion is considered the dominant phenomena, in the approximation that the effect of all other phenomena is negligible.

If the diffusion takes place only on *x*-axis, Fick's first law can be written in the form

(1) $$\begin{array}{c} J=-D\frac{\partial C}{\partial x}, \end{array}$$

where *J* is the diffusion flux, *C* the drug concentration, ∂*C*/∂*x* the drug concentration gradient, and *D* the diffusion coefficient. Assuming that the ensemble, DDS and release environment, is isolated, the concentration variation in time ∂*C*/∂*t* is numerically equal with the flux difference ∂*J*/∂*x*:

(2) $$\begin{array}{c} \frac{\partial C}{\partial t}=-\frac{\partial J}{\partial x}. \end{array}$$

By combining these equations, the diffusion equation, that is, Fick's second law, results:

(3) $$\begin{array}{c} \frac{\partial C}{\partial t}=\frac{\partial }{\partial x}D\left(\;\frac{\partial C}{\partial x}\;\right). \end{array}$$

The generalized diffusion equation, for the case of diffusion along all three axes* x, y,* and* z* can be written as [2]

(4) $$\begin{array}{c} \frac{\partial C}{\partial t}=\frac{\partial }{\partial x}D\left(\;\frac{\partial C}{\partial x}\;\right)+\frac{\partial }{\partial y}D\left(\;\frac{\partial C}{\partial y}\;\right)+\frac{\partial }{\partial z}D\left(\;\frac{\partial C}{\partial z}\;\right). \end{array}$$

Since water and drug diffusion take place simultaneously, it can be further generalized to

(5) ∂Cm∂t=∂∂xDm∂Cm∂x+∂∂yDm∂Cm∂y+∂∂zDm∂Cm∂z,

where *C*_*m*_ is the concentration and *D*_*m*_ is the diffusion coefficient for water (*m* = 1) and drug (*m* = 2).

In the particular case of a cylindrical DDS, the diffusion equation can be written under the form

(6) ∂Cm∂t=1r∂∂rrDm∂Cm∂r+∂∂θDmr∂Cm∂θ+∂∂zrDm∂Cm∂z,

where *C*_*m*_ and *D*_*m*_ have the same significance as above, *r* is the radial coordinate, *z* is the axial coordinate, *θ* is the angular coordinate, and *t* is time [3].

Crank [4] solved these equations, assuming constant diffusion coefficient and known initial and boundary conditions, offering an extensive number analytical solution, for different geometries [4].

But, in reality, and, implicitly in the case of DDS, the diffusion coefficient is influenced by time, position, and solute concentration, in which case the diffusion equations can not be solved, due to the high number of variable dependence.

To overcome this aspect, having in view the need to predict in an easier manner the drug release, empirical and semiempirical equations were used: the Higuchi [5] is as follows:

(7) $$\begin{array}{c} \frac{M\left(t\right)}{M_{\infty }}=K_{H}\cdot t^{1/2} \end{array}$$

and the Korsmeyer-Peppas [6] is as follows:

(8) $$\begin{array}{c} \frac{M\left(t\right)}{M_{\infty }}=K_{P}\cdot t^{n}, \end{array}$$

where *M*(*t*) is the released drug amount at *t*, *M*_*∞*_ is the released drug amount after very large time intervals, tending to infinity, equal, in most cases, with the drug amount loaded into polymeric matrices, *K*_*H*_ is the Higuchi diffusion constant, *K*_*P*_ is the Korsmeyer-Peppas constant, an indicator of release rate, and *n* is an exponent that depends on the shape of the polymeric matrix and can indicate the drug release mechanism. The Korsmeyer-Peppas is actually a generalization of the Higuchi equation; Higuchi equation is considered applicable in the first stage of the release (up to 20%), while Korsmeyer-Peppas equation is considered applicable in the first stage of the release up to 60%, until the equilibrium plateau is reached.

These models were confirmed by a plethora of experimental data, for all types of polymeric matrices and drugs, proving their validity and confirming the existence of

- *burst effect phase* (I) characterized by a high drug release rate (large drug amount released in a very short time), determined by high concentration gradients;
- *swelling phase *(II), in which drug release rate decreases, due the decrease of the concentration gradients;
- *equilibrium phase *(III), characterized by null concentration gradients and, implicitly, by constant released drug amount.

**Figure 1:**
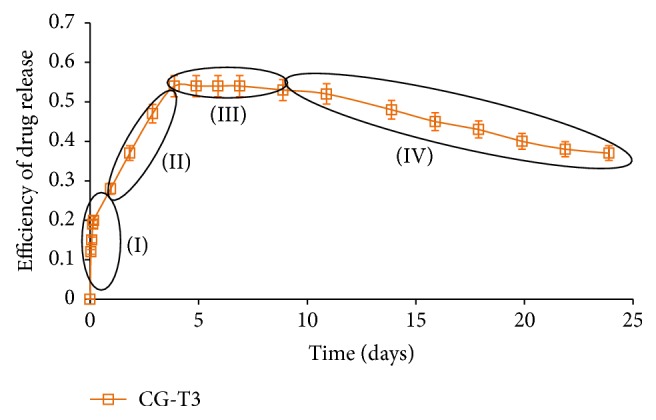
Phases demarcation in drug release mechanism [8].

In addition to these, for long time intervals, a fourth phase has been identified, namely,* degradation phase *(IV), in which polymer fragments derived from the polymer matrix bonds to released drug molecules, and, consequently, a decrease of the released drug amount is observed (Figure 1) [7, 8].

The main drawback of the above equations is that they have been demonstrated from Fick's second law, considering diffusion dominant, ignoring all other phenomena, and, moreover, assuming the release medium homogeneous and isotropic in relation to diffusion.

The use of these approximations, necessary to reduce the number of variables from the equations system that characterizes the system evolution, in order to determine its solutions, is not justified in reality. Actually, it indicates the “failure” of the classical models [1–6] and therefore the “incapacity” of some mathematical procedures based on the assumption of dynamic variables differentiability, in the analysis of complex phenomena involved in drug delivery.

However, this situation can be overcome applying nonstandard mathematical procedures based either on the nondifferentiability of the dynamic variables (remaining still tributary to differential methods) through fractal type theories or on invariance groups (any “unspecified potentiality” has the concrete expression in the existence of an invariance group).

In the present paper, we will explain the last procedure, namely, being given an “actual situation” in the form of semiempirical Higuchi law for the one-axial case; we will express “not actual” situations (potentialities) in the form of a Higuchi type law for the three-axial case, as concrete expression of Lie's group existence.

## 2. A Correlation between the One-Axial and Three-Axial Cases

The Higuchi equation is the hull of a parabolas family of the form

(9) $$\begin{array}{c} M^{2}\left(\;t\;\right)={\bar{a}}^{2}\left(\;t+\bar{b}\;\right), \end{array}$$

where $\bar{a}$ and $\bar{b}$ are constants dependent both on the external constrains (temperature, pressure, etc.) and on the structure of the polymeric matrices. In the selection of expression (9) the fact that this expression is universal was taken into account, meaning that it is valid for any polymeric matrices, no matter the shape, structure, and so on, and, also, it is valid for any “constrain” type rate, from the “burst type effect” domain to equilibrium plateau. Moreover, it involves an intrinsic isotropy, which does not correspond to reality, the drug release mechanism having a high degree of anisotropy. Still, one can establish a correlation between the one-axial case, associated with the isotropy hypothesis and the three-axial case, associated with anisotropy, as we will show next.

If we center around the parabolas family from (9), then it is clear that it must make its mark on a possible experimental plane geometry (*M*, *t*). This geometry can be founded on a parametric group which must make the form from relation (9) invariant. This group can be best revealed if the homogenous coordinates (*M*, *t*) are used in the form [9]

(10) $$\begin{array}{c} \frac{x_{1}}{x}=\frac{x_{2}}{y}=\frac{x_{3}}{1}, \end{array}$$

where

(11) x=t+b¯y=Ma¯

case in which (9) becomes

(12) $$\begin{array}{c} x_{2}^{2}-x_{1}x_{3}=0. \end{array}$$

In this situation, the conic from (12) accepts the canonic parameterization [9]:

(13) $$\begin{array}{c} \frac{x_{1}}{\tau^{2}}=\frac{x_{2}}{\tau }=\frac{x_{3}}{1}, \end{array}$$

where *τ* is a real parameter and its invariance group is the three-parameter group generated by the homographic transformation of the *τ* parameter. If this transformation is written under a more convenient form

(14) $$\begin{array}{c} \tau =\frac{\tau +\alpha_{1}}{1-\alpha_{2}-\alpha_{3}}, \end{array}$$

which highlights the unit transformation for *α*_1_ = *α*_2_ = *α*_3_ = 0, then using (13) the following transformation relations for the parameters *x*_1_ and *x*_2_ result:

(15) x1=x1+2α1x2+α12α32x1−2α31−α2x2+1−α22,x2=−α3x1+1−α2−α1α3x2+α11−α2α32x1−2α31−α2x2+1−α22,

from which a continuous two variables with three-parameter group can be observed. The Lie algebra [10] is given by the operators

(16) L1=2y∂∂x+∂∂y,L2=2x∂∂x+y∂∂y,L3=2xy∂∂x+2y2−x∂∂y,

with the commutation relations:

(17) L1,L2=L1,L2,L3=L3,L3,L1=−2L2,

where inhomogeneous coordinates were taken into account in order to simplify the writing.

As it should be, the conics in (12) appear in this situation as (16) group's invariant varieties with two parameters, and this is why they are invariant only with regard to the first two operators from (16). The issue at hand is not to find the two-parameter invariant varieties families, but to find the three-axial that holds three parameters: the main masses, that is, the eigenvalues of the mass tensor. Now the masses evolution group remains to be solved, which must be isomorphic to the group from (16). In order to highlight it we must note that the main masses are the solution to the secular equation of the respective matrix, which can be written as [9]

(18) $$\begin{array}{c} M^{3}+3a_{1}M^{2}+3a_{2}M+a_{3}=0, \end{array}$$

where 3*a*_1_, 3*a*_2_, and *a*_3_ are the orthogonal invariants of the masses matrix. If the masses state varies from *M*_1_, *M*_2_, and *M*_3_ to *M*_1_′, *M*_2_′, and *M*_3_′ then an algebra theorem [9] shows that, between the secular equations, which have the respective values as roots, a linear relation takes place, generated by the homographic transformation

(19) $$\begin{array}{c} M^{\prime }=\frac{aM+b}{cM+d} \end{array}$$

which gives a three-parameter group but in three variables. By writing the roots of the curve from relation (18) in the Barbilian form [11–13],

(20) $$\begin{array}{c} M^{\prime }=\frac{h+\varepsilon_{i}\bar{h}k}{1+\varepsilon_{i}k}, \end{array}$$

where *ε*_*i*_^3^ = 1, *h*, $\overset{-}{h}$ are quantities conjugated one to the other, *k* is a one-module complex factor, the transformation from (19) induces the quantities *h*, $\overset{-}{h}$, and *k* is the real transformations

(21) h′=ah+bch+d,h−′=ah−+bch−+d,k′=ch−+dch+dk,

which form a three variables with three-parameter group, that is, the Barbilian group [14].

This group is simple transitive, with the infinitesimal generators given by the operators [14]

(22) A1=∂∂h+∂∂h−A2=h∂∂h+h−∂∂h−A3=h2∂∂h+h−2∂∂h−+h−h−k∂∂k

which reveals for the associated Lie algebra a structure that is identical with the one from (17). Therefore the two groups are isomorphic, and operators (16) and (22) are generated by the one and the same algebra (12). Moreover, group (22), being simple transitive, is definitely measurable, its elementary measure being given by the differential three-form [14]:

(23) $$\begin{array}{c} \frac{dh\wedge d\overset{-}{h}\wedge dk}{{\left(h-\overset{-}{h}\right)}^{2}k}. \end{array}$$

Using the elementary probability

(24) $$\begin{array}{c} dP=\frac{dh\wedge d\bar{h}\wedge dk}{{\left(h-\bar{h}\right)}^{2}k} \end{array}$$

probabilities theory can be a priori constructed in the space of field variables $\left(h,\overset{-}{h},k\right)$.

This function quadratic root can be assimilated to the wave function analogue [15] and it will satisfy an equation of Schrödinger type that defines the fractal space-time geodesics [16].

The issue now at hand is to find the invariant varieties families of group (16) with three parameters, having group (22) associated as a parameters group. In our opinion these functions can provide an answer to the problem of the correlation between the one-axial behavior of the mass-time curve and the mass induced in the complex system by the one-axial experimental “strain.”

These varieties families (joint invariant functions) will be solutions of the Stoka equations [17, 18]:

(25) 2y∂f∂x+∂f∂y+∂f∂h+∂f∂h−=02x∂f∂x+y∂f∂y+h∂f∂h+h−∂f∂h−=02xy∂f∂x+2y2−x∂f∂y+h2∂f∂h+h−2∂f∂h−+h−h−k∂f∂k=0.

This system admits solutions of the form

(26) $$\begin{array}{c} f\left(\;\alpha_{0},k_{0}^{2}\;\right)=const.\;, \end{array}$$

where

(27) α0=x−y2h−h¯x−h−h¯y+hh¯k02=k2x−2yh¯+h¯2x−2yh+h2.

It can be observed that the last of these integrals is a one-module complex one. In principle, *f* can be any function which is continuous and derivable in its variables. It is not yet known what kind of interpretation can a general solution such as (26) have, but some specific integrals values from relation (27) can still be interpreted. Thus, if the experimental one-axial constrain is monotonous, then (27) must fulfill the condition *y*^2^ = *x*, fact which leads to the specific value *x* = 0. In this case, the second relation (27) gives

(28) $$\begin{array}{c} k_{0}=k\frac{y-\overset{-}{h}}{y-h}, \end{array}$$

from which we can write *y* as

(29) $$\begin{array}{c} y=\frac{\overset{-}{h}k-hk_{0}}{k-k_{0}}. \end{array}$$

The result we obtained in this case is important mainly because it shows that *y* can be identified in a specific case with one of the main masses values. Indeed, if *k*_0_ ≡ (−1, −*ε*, −*ε*^2^) then the situation from (20) is again reached. Therefore, we can state that in both these specific cases the mass in the one-axial constrain can be considered as one of the internal masses eigenvalues. However we can draw more from (29). If this equation is written for *k*_0_ = −1,

(30) $$\begin{array}{c} y=\frac{h+\overset{-}{h}k}{1+k}, \end{array}$$

and *h*, $\overset{-}{h}$, and *k* are explicitly written with regard to the main mass and also the system of (20) is solved with regard to *h*, $\overset{-}{h}$, and *k*, then the following relations can be found:

(31) h=−M2M3+εM3M1+ε2M1M2M1+εM2+ε2M3,k=M1+ε2M2+εM3M1+εM2+ε2M3.

These can be related to the above-mentioned parameters by means of relations:

(32) k=e−3iξh=13M1+M2+M3+16M1−M22+M2−M32+M1−M321/2sin⁡3ξ−icos⁡3ξ,

where

(33) $$\begin{array}{c} \tan\xi =\frac{2M_{1}-M_{2}-M_{3}}{\sqrt{3}\left(M_{2}-M_{3}\right)} \end{array}$$

is the “anisotropy” angle.

If we use (32) in (30), we obtain the following:

(34) y=13M1+M2+M3+12M1−M22+M2−M32+M1−M321/2sin⁡ξ.

The first term of (34) corresponds to the average drug mass released on the main directions, while the second term corresponds to the average drug mass released on the “mixed” directions. In other words, the first term reflects the linear evolution of the release system, that is, the dominance of individual effects and isotropy, in the first moments of the release (burst effect phase), while the second term reflects the nonlinear evolution, that is, dominance of collective effects and anisotropy, at higher time moments (swelling and equilibrium phases).

The dominance of the linear effects, which implies the functionality of the relation

(35) $$\begin{array}{c} y⟶M_{1}=\bar{a}{\left(\;t+\bar{b}\;\right)}^{1/2} \end{array}$$

results from (34) through the annulment of release system anisotropy, that is, imposing the restriction

(36) sin⁡ξ⟶0,2M1⟶M2+M3.

On the contrary, “reconsidering” the nonlinear behavior (but still remaining tributary to the linear one) through a proper choice of normalization parameters, the functionality of a relation of the following type can be induced:

(37) $$\begin{array}{c} y=A{\left(\;\tau \;\right)}^{1/2}sn\left(\;B{\left(\;\tau \;\right)}^{1/2};s\;\right), \end{array}$$

where sn is the Jacobi elliptic function of module *s* [19], *τ* is the normalized time, and *A* and *B* are constants specific for the release system. The module *s* of the elliptic function sn is a measure of system nonlinearity and, implicitly, a measure of the release degree; thus, one can also explain the release mechanism. Two extreme situations can be made explicit by the elliptic function degenerations as functions of its module values:

(38) y=A0τ1/2sin⁡B0τ1/2,for  s⟶0,y=A1τ1/2tanh⁡B1τ1/2,for  s⟶1.

**Figure 2:**
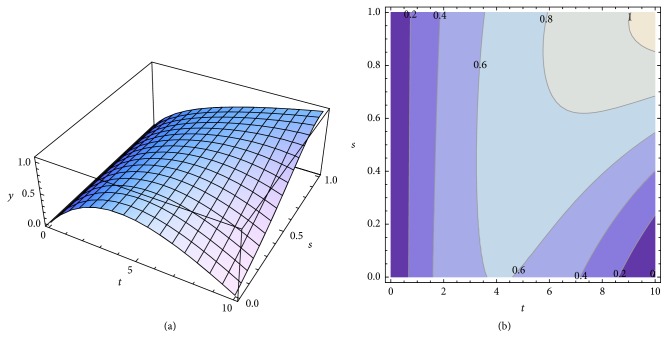
Graphical representations of relation (37), in three-dimensional format (a) and contour plot format (b).

We present the graphical representations of relation (37), in three-dimensional (Figure 2(a)) and contour plot (Figure 2(b)) formats.  Table 1 reveals the curves of equally concentration for the released drug.

## 3. Conclusions

Accepting the Higuchi semiempirical law for the one dimensional case, it is generalized to the three-axial case based on concept of joint invariant functions at the simultaneous actions of two isomorphic groups SL(2R). In this context, a new release law, independent of the polymer matrix and/or drug types, is deducted and validated by the experimental data from the literature. Thereby, also the behavior of biological structures is explained, with a high nonlinear character, under the action of drug delivery systems [20–27].

## Figures and Tables

**Table 1:** Plane sections through 3D plot, for different values of system nonlinearity *s*, can be linked to experimental drug release kinetics, that is, for different drug release mechanisms [7].

Plane sections	Experimental drug release kinetics	Release phase/drug release mechanism
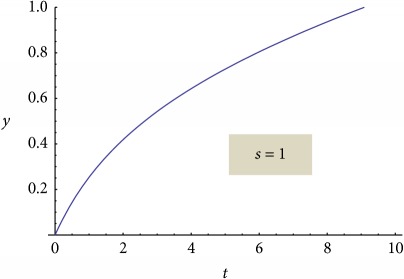	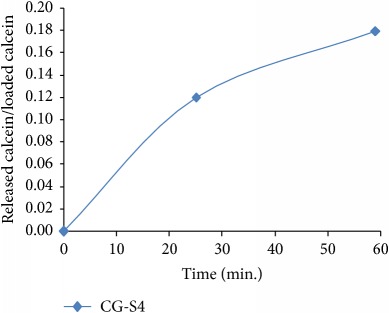	Burst effect phase/diffusion due to concentration gradient [7, 8, 28–43]
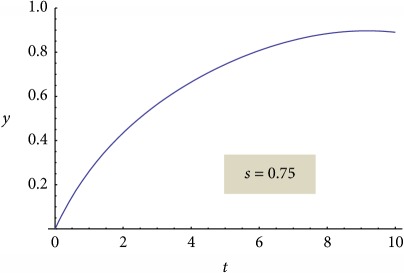	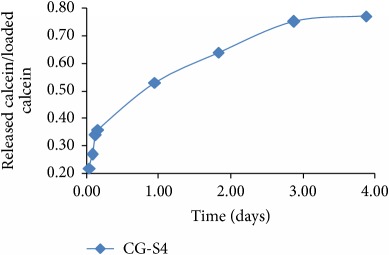	Swelling phase + equilibrium phase/drug diffusion and polymer relaxation [7, 8, 28, 30, 38, 39]
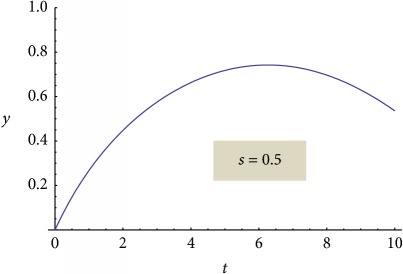	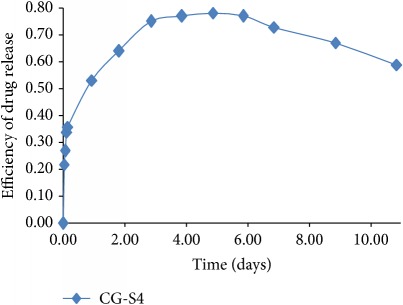	Polymer degradation phase/drug and polymer degradation, physical and chemical interactions between the resulting fragments [7, 8]

## References

[1] Siepmann J., Siepmann F., Siepmann J., Siegel R., Rathbone M. (2012). Swelling controlled drug delivery systems. *Fundamentals and Applications of Controlled Release Drug Delivery*.

[2] Siepmann J., Siegel R., Siepmann F., Siepmann J., Siegel R., Rathbone M. (2012). Diffusion controlled drug delivery systems. *Fundamentals and Applications of Controlled Release Drug Delivery*.

[3] Siepmann J., Siepmann F. (2008). Mathematical modeling of drug delivery. *International Journal of Pharmaceutics*.

[4] Crank J. (1975). *The Mathematics of Diffusion*.

[5] Higuchi T. (1963). Mechanism of sustained-action medication: theoretical analysis of rate of release of solid drugs dispersed in solid matrices. *Journal of pharmaceutical sciences*.

[6] Korsmeyer R. W., Gurny R., Doelker E., Buri P., Peppas N. A. (1983). Mechanisms of solute release from porous hydrophilic polymers. *International Journal of Pharmaceutics*.

[7] Bacaita E. S., Ciobanu B. C., Popa M. (2014). Phases in the temporal multiscale evolution of the drug release mechanism in IPN-type chitosan based hydrogels. *Physical Chemistry Chemical Physics*.

[8] Bacaita E. S., Agop M. (2016). Multiscale mechanism of drug release from polymeric matrices: a confirmation through a nonlinear theoretical model. *Physical Chemistry Chemical Physics*.

[9] Mihăileanu N. (1972). *Complements of Geometry: Analytical, Projective and Differential*.

[10] Duistermaat J. J., Kolk J. A. C. (2000). *Lie Groups*.

[11] Barbilian D. (1938). *Riemannsche Raum Cubisher Binar Former*.

[12] Barbilian D. (1971). *Elementary Algebra, The Didactic Works of Dan Barbilian*.

[13] Barbilian D. (1974). *Geometry and the Theory of Functions, The Didactic Works of Dan Barbilian*.

[14] Mazilu N., Agop M. (2012). *Skyrmions. A Great Finishing Touch to Classical Newtonian Philosophy*.

[15] Jaynes E. T. (1973). The well-posed problem. *Foundations of Physics. An International Journal Devoted to the Conceptual Bases and Fundamental Theories of Modern Physics, Biophysics, and Cosmology*.

[16] Merches I., Agop M. (2016). *Differentiability and Fractality in Dynamics of Physical Systems*.

[17] Stoka M. I. (1967). *Integral Geometry*.

[18] Stoka M. I. (1968). *Geometrie Integrale, Memorials des Sciences Mathematiques*.

[19] Bowman F. (1953). *Introduction to Elliptic Functions with Applications*.

[20] Postolache P., Petrescu V., Dumitrascu D. D., Rimbu C., Vrînceanu N., Cipaian C. R. (2016). Research Regarding a Correlation Core–Shell Morphology–Thermal Stability of Silica–Silver Nanoparticles. *Chemical Engineering Communications*.

[21] Postolache P., Cozma C. D., Cojocaru D. C. (2013). Assessment of nicotine dependence in a large cohort of smokers - social and medical aspects. *Revista de Cercetare si Interventie Sociala*.

[22] Postolache P., Duceac L. D., Vasincu E. G., Agop M., Nemeş R. M. (2016). Chaos and self-structuring behaviors in lung airways. *Scientific Bulletin-University Politehnica of Bucharest*.

[23] Nemeș R. M., Duceac L. D., Vasincu E. G., Agop M., Postolache P. (2015). On the implications of the biological systems fractal morpho-functional structure. *Scientific Bulletin-University Politehnica of Bucharest*.

[24] Paun V. A., Popa M., Desbrieres J. (2016). Liposome loaded chitosan hydrogels, a promising way to reduce the burst effect in drug release. A comparativ analysis. *Revista de Materiale Plastice*.

[25] Paun V. A., Ochiuz L., Hortolomei M. (2016). In vitro release kinetics evaluation of erythromycin in microemulsions for dermal applications. *Revista de Materiale Plastice*.

[26] Nemes R. M., Ianosi E. S., Pop C. S. (2015). Tuberculosis of the oral cavity. *Romanian Journal of Morphology and Embryology*.

[27] Știrbu I., Vizureanu P., Cimpoeșu R. (2015). Advanced metallic materials response at laser excitation for medical applications. *Journal of Optoelectronics and Advanced Materials*.

[28] Peptu C. A., Ochiuz L., Alupei L., Peptu C., Popa M. (2014). Carbohydrate based nanoparticles for drug delivery across biological barriers. *Journal of Biomedical Nanotechnology*.

[29] Bungău S., Bungău C., Ţiţ D. M. (2015). Studies about last stage of product lifecycle management for a pharmaceutical product. *Journal of Environmental Protection and Ecology*.

[30] Cojocaru I. L., Ochiuz L., Spac A. (2012). The validation of the UV spectrophotometric method for the assay of 5 fluorouracil. *Farmacia*.

[31] Bungǎu S., Szabo I., Badea G. E., Fodor A. (2012). Biotin determination by using a kinetic method. *Revue Roumaine de Chimie*.

[32] Copolovici L., Bungău S., Drăgan F. (2005). Determination of acetylsalicylic acid from drugs using a kinetic method. *Revista de Chimie*.

[33] Bungău S., Drăgan F., Moldovan A. (2005). Synthesis and analysis of ion association complexes of metoprolol. *Revista de Chimie*.

[34] Drăgan F., Bungău S., Moldovan A. (2005). Synthesis and analysis of ion association complexes of atenolol. *Revista de Chimie*.

[35] Bungău S., Copolovici L., Bâldea I., Szabo I. (2004). Determination of methionine from pharmaceutical products using the oxidation reaction with potassium permanganate. *Revista de Chimie*.

[36] Bungău S., Bâldea I., Copolovici L. (2003). Determination of ascorbic acid in fruit using a Landolt type method. *Revista de Chimie*.

[37] Bungău S., Copolovici L., Bâldea I., Merca V. (2004). Pyridoxine determination of pharmaceuticals by using two kinetic methods. *Revista de Chimie*.

[38] Grigoras A. G., Constantin M., Grigoras V. C., Dunca S. I., Ochiuz L. (2013). Studies on physico-chemical and antibacterial properties of grafted pullulans solutions. *Reactive and Functional Polymers*.

[39] Ochiuz L., Stanescu A., Stoleriu I. (2011). Investigations on the in vitro release mechanism of propiconazole nitrate from hydrophilic gel formulations. *Farmacia*.

[40] Fodor A., Petrehele A. I. G., Bota S. R., Bungău S. (2011). Hydrothermal synthesis and comparative study of two silicium monolacunar polyoxometalates Zn complexes K7[Zn(SiM_10_VO_39_)(H_2_O)].nH_2_O (M=Mo,W). *Revista de Chimie*.

[41] Petrehele A., Fodor A., Bungău S. (2010). Rational synthesis, characterisation and crystal structure study of Keggin monolacunar arsenate(V)-vanadium(V)-tungsteno(VI) Zn(II) complex. *Revista de Chimie*.

[42] Jurca T., Bănică F., Bungău S. (2006). The study of the mass spectra of pyrazinamide and its complex combinations with copper(II) benzoate. *Revista de Chimie*.

[43] Cărăban A., Bungău S., Fodor A., Stănăşel O. (2006). Influence of ascorbic acid, thiamine and riboflavin on starch hydrolysis endogenous amylase using laser interferometry. *Revista de Chimie*.

